# The influence of variations in actual evapotranspiration on drought in China's Southeast River basin

**DOI:** 10.1038/s41598-023-48663-8

**Published:** 2023-12-04

**Authors:** Sheng Hong, Haijun Deng, Zhouyao Zheng, Yu Deng, Xingwei Chen, Lu Gao, Ying Chen, Meibing Liu

**Affiliations:** 1 Institute of Geography, Fujian Normal University, Fuzhou, 350117 China; 2 Fujian Provincial Engineering Research Centre for Monitoring and Assessing Terrestrial Disasters, Fujian Normal University, Fuzhou, 350117 China; 3 Key Laboratory of Humid Subtropical Eco-Geographical Processes of Ministry of Education, School of Geographical Sciences, Fujian Normal University, Fuzhou, 350117 China; 4 Fujian Provincial Key Laboratory for Plant Eco-Physiology, Fujian Normal University, Fuzhou, 350117 China

**Keywords:** Climate sciences, Hydrology, Natural hazards

## Abstract

Revealing changes in actual evapotranspiration is essential to understanding regional extreme hydrological events (e.g., droughts). This study utilized the Global Land Evaporation Amsterdam Model (GLEAM) to analyse the spatial and temporal characteristics of actual evapotranspiration over 40 years in the Southeast River basin of China. The relationship between changes in actual evapotranspiration and the drought index was quantified. The results indicated a significant increase in actual evapotranspiration in the Southeast River basin from 1981 to 2020 (2.51 mm/year, *p* < 0.01). The actual evapotranspiration components were dominated by vegetation transpiration (73.45%) and canopy interception (18.26%). The actual evapotranspiration was closely related to the normalised difference vegetation index (r = 0.78, *p* < 0.01), and vegetation changes could explain 10.66% of the increase of actual evapotranspiration in the Southeast River basin since 2000. Meanwhile, actual evapotranspiration and standardised precipitation evapotranspiration index (SPEI) showed a highly significant negative spatial correlation, with a Moran's I index of − 0.513. The rise in actual evapotranspiration is an important trigger factor for seasonal droughts in the region. Therefore, these results help deepen the understanding of hydro-climatic process changes in the southeastern coastal region of China.

## Introduction

Evapotranspiration is a crucial hydrological process that connects water, soil, vegetation, and atmosphere^1^. Actual evapotranspiration (ET_a_) includes evaporation from the soil and open water surfaces and transpiration through the plant canopy. It accounts for approximately 59% of terrestrial precipitation^2^. The changing characteristics of ET_a_ and its potential impacts are at the forefront of climate change research^3–5^.

ET_a_ in the southeastern coastal region of China has increased significantly (1.87 mm/year) during the past four decades^6^. This region has experienced a temperature increase of approximately 0.03 °C/year since 2000^7^. Simultaneously, this region experienced one of China’s most significant upward trends in annual precipitation during 1990–2019, with an increase of approximately 5.9 mm/year^8^. The rising ET_a_ in this region may be due to the combined effects of temperature and precipitation. In general, ET_a_ changes in humid regions are energy limited as they are related to changes in temperature or wind speed^9,10^. Current studies have mainly focused on the temporal and spatial variations in ET_a_ in the southeastern coastal region of China^11,12^. It is worth noting that vegetation changes significantly impact ET_a_^13^. Transpiration through plant canopy constitutes 57.2% ± 6.8% of global evapotranspiration^14^, representing a key component of ET_a_. For example, in the Haihe River basin, forest land is equivalent to only 38.57% of cultivated land, but ET_a_ in the forest is approximately 81 mm higher than those in cultivated land^14^. In the eastern monsoon region of China, vegetation transpiration is dominated by ET_a_, which accounts for more than 70% of the total ET_a_; therefore, changes in vegetation need to be considered when attributing changes in ET_a_^15^. Rapid land-use changes in the southeastern coastal region of China, ET_a_, and the evolution of component proportions are important elements of the regional hydrological cycle^16^. Research on the variability of ET_a_ components in this region and their impacts is still in its infancy^7,9^.

Previous studies have indicated that increases in ET_a_ lead to regional drought events by influencing soil moisture deficits^17,18^. For example, a past study showed that an increase in ET_a_ accelerated the formation and expansion of summer droughts in Europe^11,12^. Seneviratne et al. determined that ET_a_ was the most important driving factor in the early drought stage but its role gradually weakened with the intensification of drought^19^. In the southeastern coastal region of China, the drought frequency increased while ET_a_ increased^20^. However, the relationship between evapotranspiration and drought has been little studied and has not yet been determined^21–23^.

Therefore, this study selected the Southeast River basin as the study area and used Global Land Surface Evaporation: Amsterdam Methodology (GLEAM) data to analyse ET_a_ changes and their influencing factors from 1981 to 2020. The standardised precipitation evapotranspiration index (SPEI) data were then used to explore the correlation between changes in ET_a_ and drought. The remainder of the study is structured as follows: Section “Materials and methods” outlines the “Materials and methods” used in the study; Section “Results” analyses ET_a_ changes and influencing factors, and the relationship between ET_a_ and droughts; Section “Discussion” section provides a comprehensive discussion; finally, Section “Conclusion” presents the concluding remarks.

## Materials and methods

### Study area

**Figure 1:**
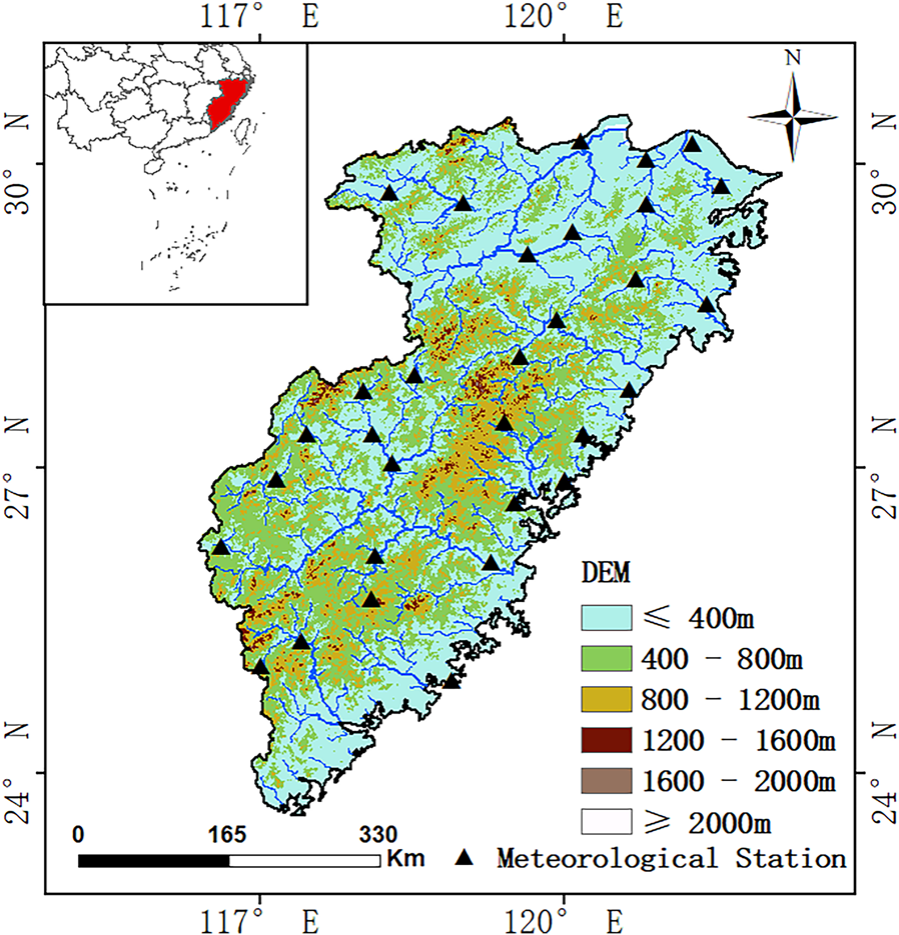
The location map of the Southeast River basin of China. (We used ArcGIS10.2 to generate the figure).

The Southeast River basin is located in the southeastern coastal region of China (Fig. 1). The basin covers the provinces of Fujian, Zhejiang, Anhui, and Taiwan and is one of the most important water resource subdivisions in China. Because of the lack of data for Taiwan, the scope of the Southeast River basin investigated in this study was limited to mainland China. The watershed covers an area of 24.06 × 10^4^ km^2^ and is situated between 21°90′N ~ 30°42′N and 116°40′E ~ 122°14′E. The region features a subtropical monsoon climate with an average annual temperature of 18.10 °C and an annual precipitation of 1624.06 mm. The Southeast River basin has experienced a temperature increase of approximately 0.03°C/year over the past two decades and has witnessed one of China's most significant upward trends in annual precipitation (5.9 mm/year)^7,8^. Although the Southeast River basin has a typical subtropical monsoon climate, it is subject to drought events and is less resilient to drought risk.

### Data sources

#### Meteorological data

The meteorological data utilised in this study were sourced from the "Daily Value Data Set of Chinese Terrestrial Climate Information (V3.0)" provided by the National Meteorological Information Center's, China Meteorological Administration (https://data.cma.cn/, accessed on 22 July 2023). The dataset comprised 31 meteorological stations in the Southeast River basin (Fig. 1). This study collected daily meteorological data from 31 stations spanning 1981–2020, encompassing mean temperature (℃), maximum temperature (℃), minimum temperature (℃), wind speed (m/s), relative humidity (%), and precipitation (mm).

#### GLEAM data

The GLEAM dataset is a commonly used remote-sensing-based actual evapotranspiration product developed and provided by the University of Amsterdam (https://www.gleam.eu/, accessed 22 July 2023)^9,24,25^. There are five major remote sensing products for ET_a_: GLEAM, the Breathing Earth System Simulator (BESS), the Advanced Very High-Resolution Radiometer (AVHRR), the Moderate-Resolution Imaging Spectroradiometer 16 (MOD16), and the Operational Simplified Surface Energy Balance (SSEBop)^26–30^. Among these, GLEAM, BESS, and AVHRR are more accurate in the wet region, while BESS has the largest average bias at the monthly scale (− 8.34 mm), which indicates a large systematic error; AVHRR has a higher accuracy at the global scale but a larger mean deviation of 114.05 mm/year at the basin scale, which is not applicable to the study area; MOD16 and SSEBop products are more suitable for arid and semi-arid regions, which is not consistent with the climatic characteristics of the Southeast River basin^31^. The advantage of the GLEAM product over the other four products is that it is suitable for cloudy conditions and has a higher accuracy in the Southeast River basin, with a deviation of − 1.60, which is suitable for this study area^32^. Therefore, the GLEAM product was selected for the study of temporal and spatial changes and factors influencing ET_a_.

The GLEAM product utilised in this study exhibited a spatial resolution of 0.25° and a temporal resolution of 1 d for 1981–2020. The GLEMA ETa comprises five primary constituent components^27,33–35^: transpiration (Et), forest canopy interception (Ei), water evaporation (Ew), bare-ground evaporation (Eb), and snow sublimation (Es). The amount of Es was negligible because of the low latitude of the Southeast River basin, and minimal seasonal snow accumulation occurred only in the local northern area. Hence, Et, Ei, Ew, and Eb were analysed in this study.

**Figure 2:**
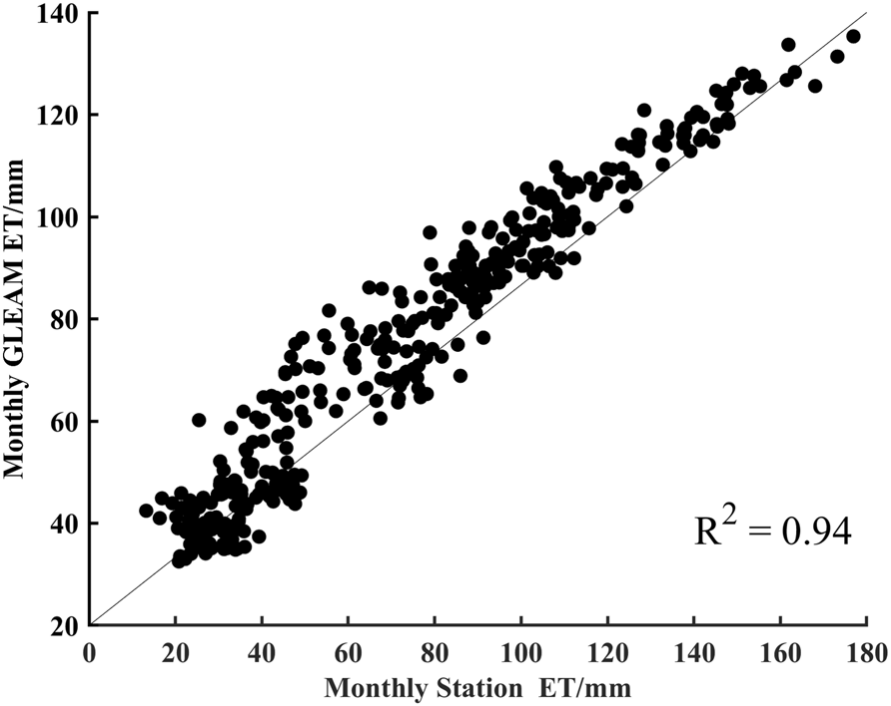
Comparison of GLEAM ET_a_ with monthly station P-M ET_a_ in the Southeast River basin during the period from 1983 to 2013.

Figure 2 shows a good correspondence between the GLEAM ET_a_ and the Penman–Monteith model (P-M) ET_a_ of the stations. The Nash–Sutcliffe efficiency coefficient (NSE), coefficient of linear regression determination (R^2^), and percentage deviation (P_bias_) were used to assess the suitability of the GLEAM ET_a_ data. The closer NSE and R^2^ are to 1 and the closer P_bias_ is to 0, the more suitable the GLEAM ET_a_ data. The results showed that the GLEAM ET_a_ data are suitable for application in Southeast River basins, with NSE, R^2^, and P_bias_ values of 0.87, 0.94, and − 1.60% respectively. Therefore, this study utilised GLEAM v3.5a ET_a_ data to examine the spatiotemporal characteristics and components of ET_a_ in the Southeast River basins.

#### NDVI index

This study used normalised difference vegetation index (NDVI) data from the Geographic Remote Sensing Ecological Network platform (http://www.gisrs.cn/, accessed on 22 July 2023). This dataset was based on a continuous time series of SPOT/VEGETATION NDVI satellite remote sensing data using the maximum value synthesis method to generate an annual NDVI dataset from 1981 to 2020 with a resolution of 30 m.

#### Soil moisture data

The soil moisture data used in this study were sourced from the ERA5-Land dataset (https://cds.climate.copernicus.eu/; accessed 22 July 2023). Specifically, the ERA5-Land dataset was employed to compute regional soil moisture content at a spatial resolution of 0.1° and temporal resolution of 1 h, from 1981 to 2020. This comprehensive dataset offers four distinct layers of soil thickness, at depths ranging from 0 to 7, 7 to 28, 28 to 100, and 100 to 289 cm.

#### Drought index

This study employs SPEI data sourced from the SPEI base v.2.7 (https://spei.csic.es/, accessed on 22 July 2023). This index efficiently identified various types of droughts and their impacts in the context of global warming. The dataset is highly applicable in wet areas^36,37^, with positive values indicating regions with higher moisture content and negative values representing dry regions. The dataset had a temporal resolution of monthly, spatial resolution of 0.5°, and time series from 1981–2020.

### Statistical methods

#### Mann–Kendall (M–K) nonparametric tests

The Mann–Kendall (M–K) nonparametric test is a time-series analysis method commonly used to analyse climate and hydrological changes^38–40^. In this study, the M–K trend test method was employed to calculate trends in ET_a_, and Sen's estimation method was used to estimate the trend rates^41,42^. The M–K mutation test was used to test for mutation points in ET_a_.

#### Multiple regression model

Multiple regression analysis was used to analyse the quantitative relationship between the respective variables and the dependent variable in the context of linear correlation to obtain the level of response of the independent variable to changes in the dependent variable. In this study, multiple regression analysis was employed to examine the impact of the influencing factors on ET_a_ changes^43^. As the dependent variable, ET_a_ data and its corresponding variables, including air temperature, wind speed, relative humidity, and NDVI data, were standardised to ensure that all data fell within the range of 0–1. Subsequently, multiple regression analyses were conducted for each variable to derive regression coefficients for the standardised data series. The contribution of each variable to a change in the dependent variable was calculated using the following equation:

1 $${Y}_{s}={aX}_{1}+{bX}_{2}+\dots +{cX}_{i}$$

2 $$\mu =\frac{{a\Delta X}_{1}}{{\Delta Y}_{s}}$$

where ${Y}_{s}$ is the standardised value of the dependent variable, ${X}_{1}$, ${X}_{2}$, …, ${X}_{i}$ is the standardised value of the independent variable, $a$, $b$, $c$ … are the regression coefficients after standardisation of the data series, $\mu$ is the actual contribution to the change in ${Y}_{s}$ when ${X}_{1}$ changes, ${\Delta X}_{1}$ is the change in ${X}_{1}$, and ${\Delta Y}_{s}$ is change in ${Y}_{s}$.

#### Moran’s Index

The global Moran’s Index and the local Indicators of Spatial Association (LISA) can well reflect the spatial correlation of the variables. This study used these two methods to explore whether ET_a_ affects soil moisture and drought in the Southeast River basin. The Moran’s Index was proposed by the Australian statistician Parker Moran in 1950, with a range of [− 1,1]^44^. The closer the Moran’s Index is to − 1, the stronger the negative correlation of the elements in space, and the closer it is to 1, the stronger the positive correlation. LISA was divided into four clusters of “high-high”, “low-low”, “low-high”, and “high-low”, which were represented by the attributes of one factor in the region and the influence between it and another factor^45^. When the “low-high” and “high-low” zones were larger, the negative correlation of the region was more prominent. In this study, the Moran’s Index (Eq. (3)) and LISA (Eq. (4)) calculations were performed using the Geoda software.

3 $$I=\frac{n}{{S}_{o}}\frac{\sum_{i=1}^{n}\sum_{j=1}^{n}w_{i,j}{z}_{i}{z}_{j}}{\sum_{i=1}^{n}{z}_{i}^{2}}$$

4 $${I}_{i}=\frac{{Z}_{i}}{{{S}_{o}}^{2}}\sum_{j\ne 1}^{n}{w}_{ij}{Z}_{j}$$

where ${z}_{i}$ is the deviation of the attribute of the element from its mean, $w_{ij}$ is the spatial weight between elements $i$ and $i$, $n$ is the total number of elements, and ${S}_{o}$ is the aggregation of all spatial weights.

## Results

### ET_a_ changes in the Southeast River basin

#### Temporal changes

**Figure 3:**
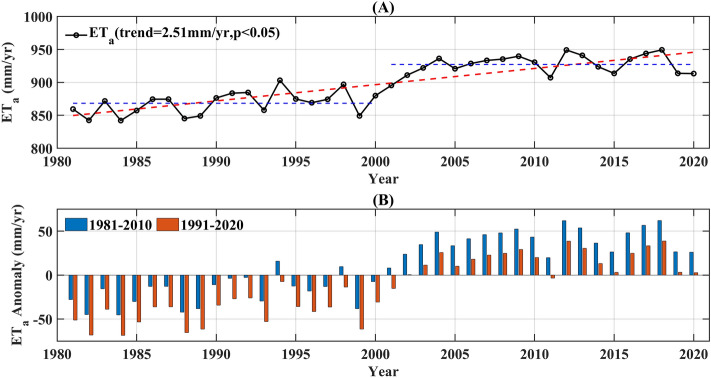
Change in annual ET_a_ from 1981 to 2020: (**A**) annual ET_a_; (**B**) ET_a_ change. Blue bars indicate the base period 1981–2010 red bars indicate the base period 1991–2020.

From 1981 to 2020, the Southeast River basin experienced a minimum annual ET_a_ of 842.04 mm in 1984 and maximum value of 949.34 mm in 2012. ET_a_ exhibited a significant increasing trend (Fig. 3A) over this period, with an acceleration rate of 2.51 mm/year (*p* < 0.05). Figure 3A demonstrates a relatively substantial increase in ET_a_ from 1981 to 2020, with the average ET_a_ increasing from 868.29 mm for 1981–2000 to 927.16 mm for 2001–2020, with a transition from negative to positive ET_a_ anomalies occurring in the year 2000 (refer to Fig. 3B). The M–K mutation test also indicated an abrupt change in ET_a_ around 2000 (Fig. S1). This revealed large differences in annual ET_a_ in the southeastern river basins and a significant increase after 2000.

**Figure 4:**
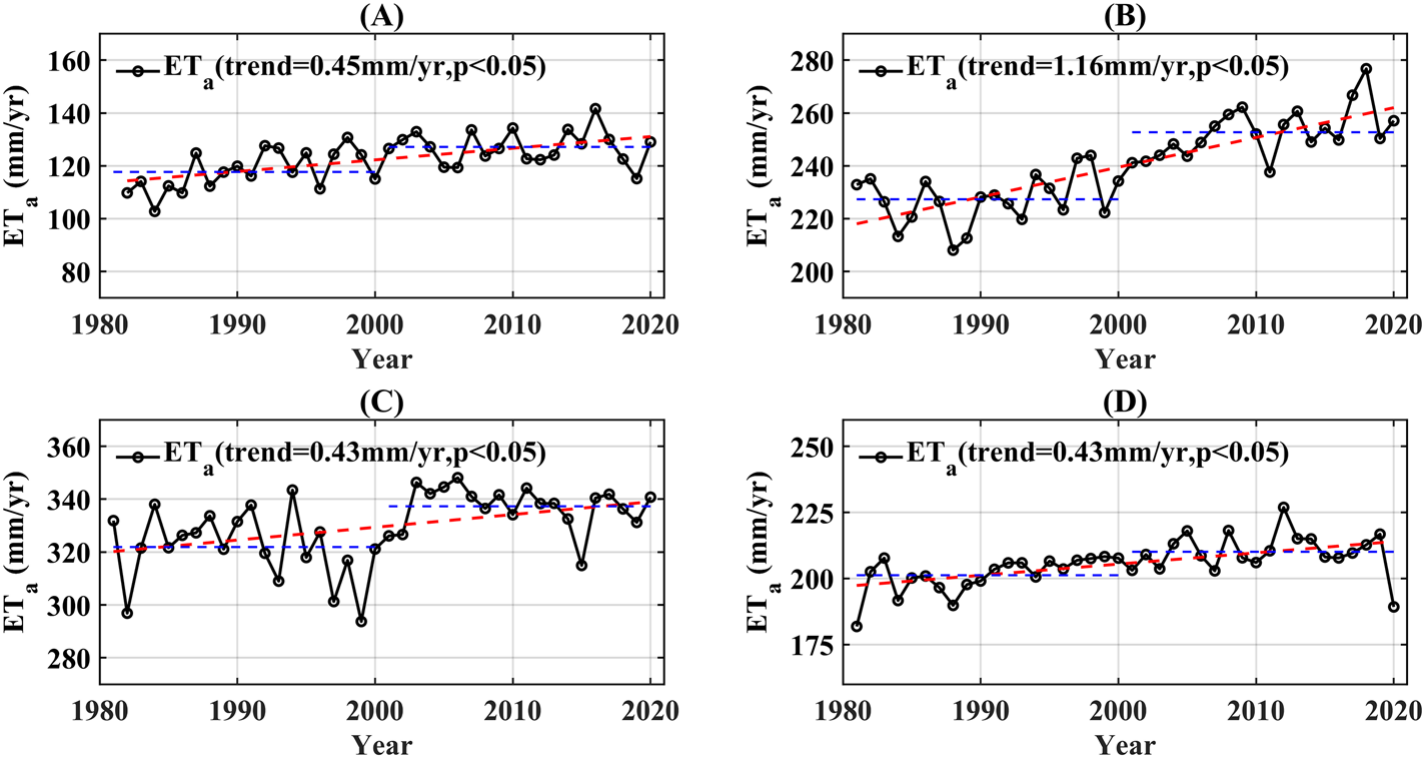
Seasonal variation of ET_a_ in the Southeast River basin from 1981 to 2020. (**A**) winter, (**B**) spring, (**C**) summer, and (**D**) autumn.

Meanwhile, ET_a_ variation exhibited significant seasonal disparities within the Southeast River basin. During the winter, the average ET_a_ was 122.43 mm, ranging from 102.74 mm in 1984 to 141.64 mm in 2016 (Fig. 4A). Overall, there was a significant increasing trend in the winter ET_a_ (0.45 mm/year, *p* < 0.05). In spring, the average ET_a_ was 240.03 mm, with the highest recorded value of 276.77 mm in 2018 and lowest of 208.05 mm in 1988 (Fig. 4B). The most significant increase in ET_a_ was observed during spring, at a rate of 1.16 mm/year (*p* < 0.05). During the summer, the average ET_a_ was 329.61 mm, with an increasing trend of 0.43 mm/year (*p* < 0.05). There was a maximum value of summer ET_a_ in 2006 of 348.12 mm and a minimum value of 293.72 mm in 1999 (Fig. 4C). The autumnal increase in ET_a_ was comparable to that of summer, with an average total ET_a_ of 205.67 mm. The highest value was recorded in 2012 at 226.90 mm and the lowest was recorded in 1981 at 181.86 mm (Fig. 4D). A comparison of the average annual ET_a_ for each season between 1981–2000 and 2001–2020 revealed that the most significant change occurred in spring during the latter period, with the increase in ET_a_ during spring contributing 43% of the multiyear change in ET_a_, followed by summer (26%), winter (16%), and autumn (15%). Over the past four decades, the increase in ET_a_ in the Southeast River basin has been primarily attributed to the spring and summer ET_a_ increase.

#### Spatial variations

**Figure 5:**
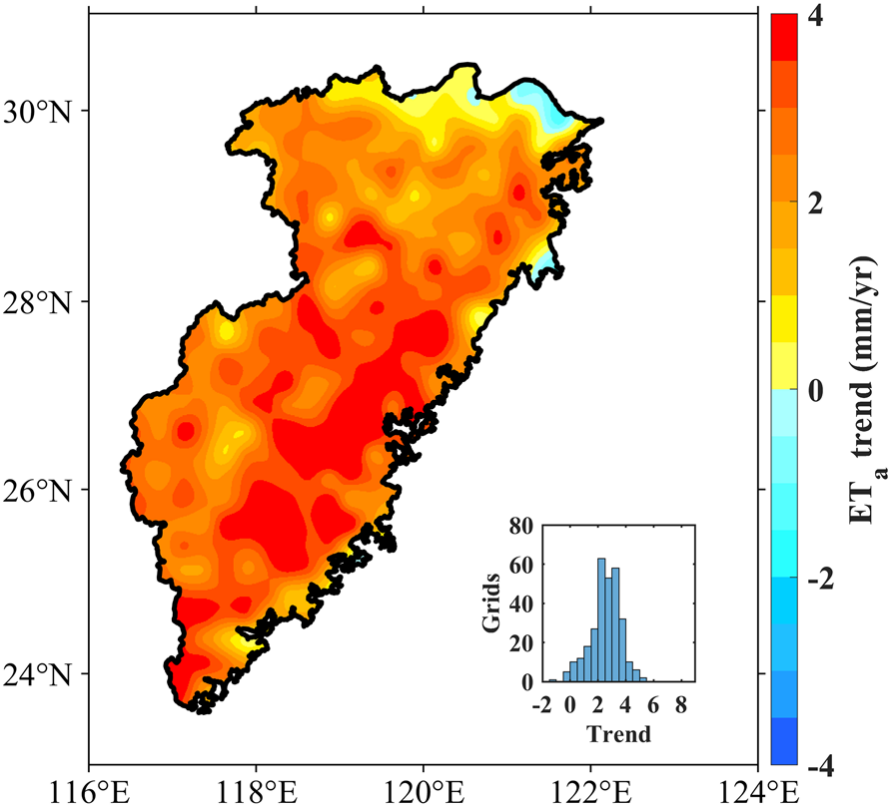
Spatial distribution of annual changes in ET_a_ from 1981 to 2020. (We used MATLAB2021a to generate the figure, https://ww2.mathworks.cn/products/matlab.html).

The spatial variability in the multiyear average ET_a_ in the Southeast River basins exhibited significant differences, ranging from 534.63 to 1345.96 mm. As shown in Fig. 5, ET_a_ in most regions exhibited a dominant increasing trend, with a decreasing trend of ET_a_ only occurring in the northern part. The histogram indicates that most grids experienced an ET_a_ increase rate ranging from 1.50 to 3.50 mm/year, which corresponds well with the spatial pattern of ET_a_.

**Figure 6:**
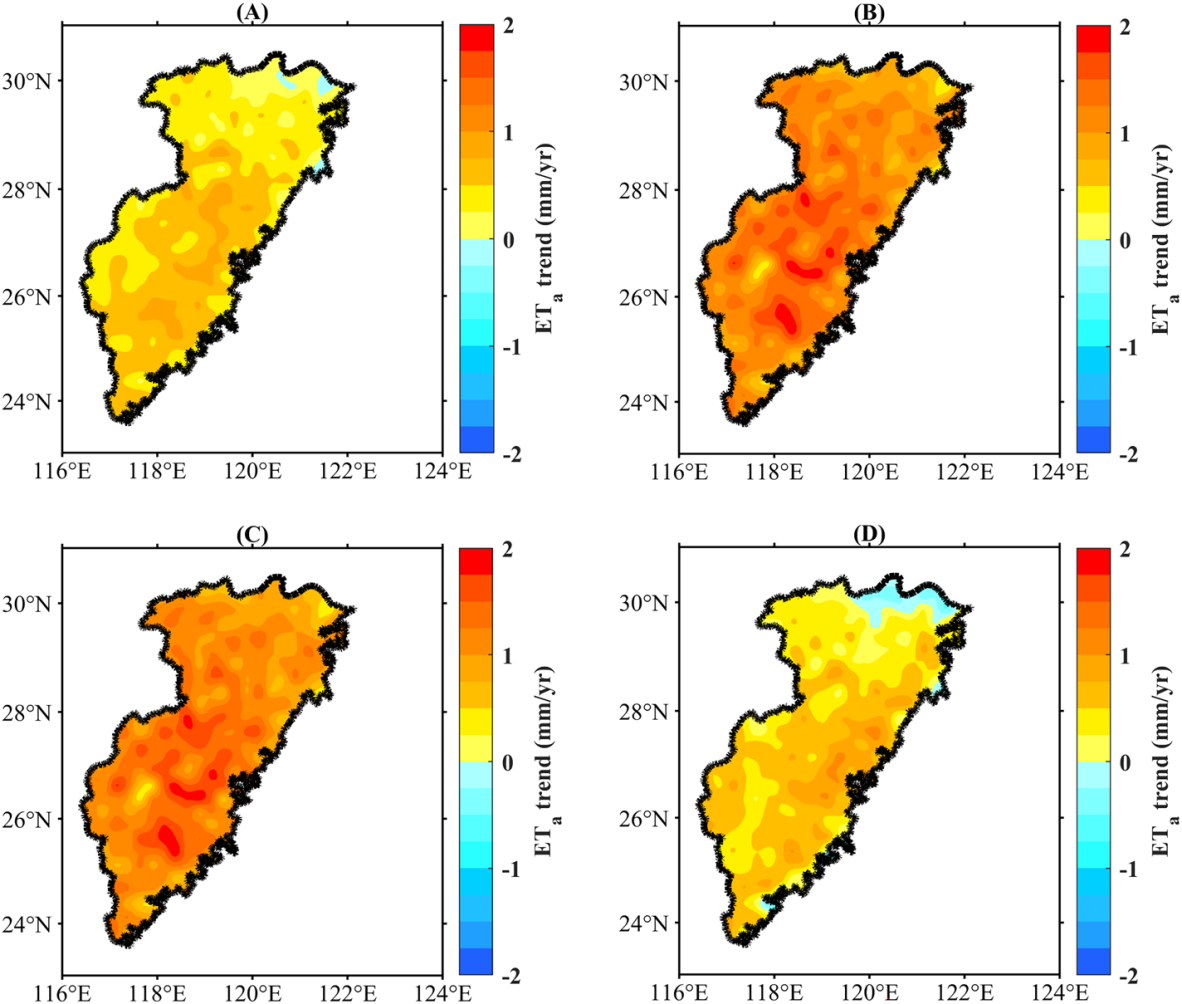
Spatial variations of seasonal ET_a_, (**a**) winter, (**b**) spring, (**c**) summer, (**d**) winter. (We used MATLAB2021a to generate the figure, https://ww2.mathworks.cn/products/matlab.html).

The spatial variation in ET_a_ in the basin of the Southeast River showed significant seasonal differences. In winter, the basin-wide ET_a_ tended to increase, except at the individual grid points; however, the rate of increase was relatively low, at less than 1 mm/year (Fig. 6A). Notably, the southern region exhibited a faster rate of increase than its northern counterpart owing to higher temperatures during winter. In most areas, the ET_a_ rate increased by 1.0–2.0 mm/year during spring (Fig. 6B). The central part of the basin experienced a higher rate of increase than other regions. In summer, the basin experienced an increase in ET_a_, with a rate of over 1 mm/year for most grids (Fig. 6C), similar to that in spring (Fig. 6B). During autumn, the central and southern regions of the basin showed a faster increase in ET_a_ than other areas; however, some parts of the northern region witnessed a lower rate of increase or even decrease (Fig. 6D). However, all four seasons showed lower growth rates in the northern region than in the other regions. This may be attributed to the lower elevation of the region and greater increase in urban land use, resulting in a lower increase in ET_a_^17^.

#### *ET*_*a*_* components changes*

The predominant contributor to ET_a_ in the Southeast River basin was Et, which accounted for 73.45%, followed by Ei, at 18.26%. Over the past four decades, Et, Ei, Ew, and Eb in the Southeast River basin have exhibited increasing trends (Table 1), with Ei, Ew, and Eb passing the significance test at the 99% confidence level. By comparing the changes in ET_a_ between 1981–2000 and 2001–2020, a significant increase of 58.87 mm was observed in ET_a_ during the latter period compared to the former. This increase can be attributed to the Ei (18.49 mm; 31.41%) and Et (27.25 mm; 46.28%). Both Ew and Eb exhibited increasing trends, albeit relatively small. Therefore, the primary contributors to the increase in ET_a_ in the Southeast River basin over the past 40 years were Et and Ei; thus, vegetation is one of several influential factors that warrant attention.

**Table 1:** Multi-year mean values and trends of each component of ET_a_ in the basin.

Component	1981–2020 (mm)	T1: 1981–2000 (mm)	T2: 2001–2020 (mm)	T2-T1	Trend (mm/year )
ET_a_	897.73	868.29	927.16	58.87	2.51**
Et	659.36	645.77	673.02	27.25	0.87
Ei	163.97	154.73	173.22	18.49	0.93**
Ew	51.33	50.07	52.59	2.52	0.09**
Eb	19.27	17.72	20.83	3.11	0.13**

**Indicates significance at a 95% confidence level.

**Figure 7:**
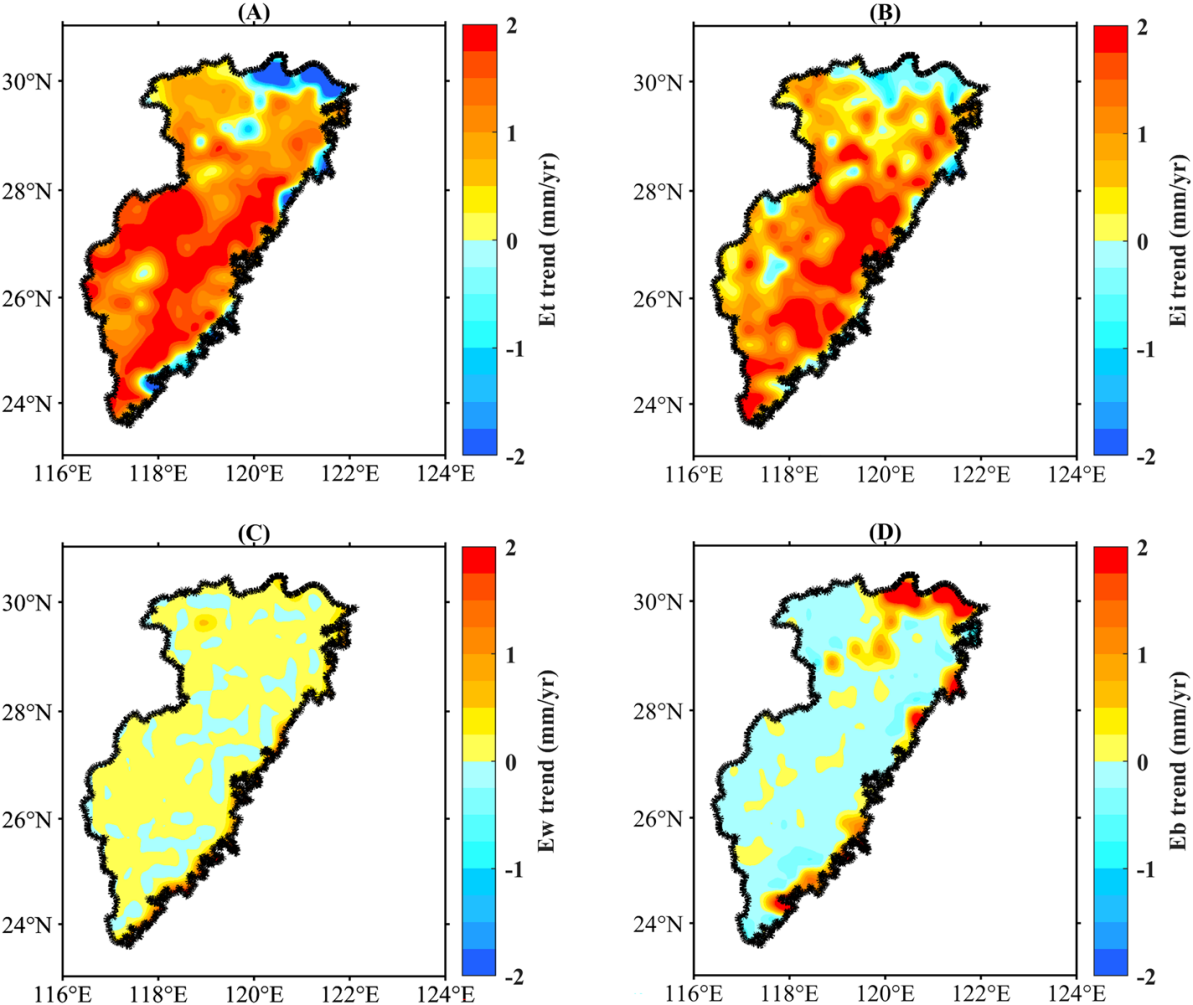
Spatial variations of ET_a_ components: (**A**) Et, (**B**) Ei, (**C**) Ew, (**D**) Eb. (We used MATLAB2021a to generate the figure, https://ww2.mathworks.cn/products/matlab.html).

Et showed significant growth in most of the basin and a negative growth trend appeared only in the northern and eastern coastal regions (Fig. 7A). The regional variation in Ei was similar to but smaller than that in Et (Fig. 7B). Figure 7C shows that the changes in Ew were relatively dispersed. The distributions of Eb were opposite to those of Et and Ei, forming a nearly complementary spatial pattern (Fig. 7D). In general, the change in the distribution of Et and Ei was consistent with that of ET_a_, which further indicates that Et and Ei are the main contributing factors to the growth of ET_a_ in the Southeast River basin.

**Figure 8:**
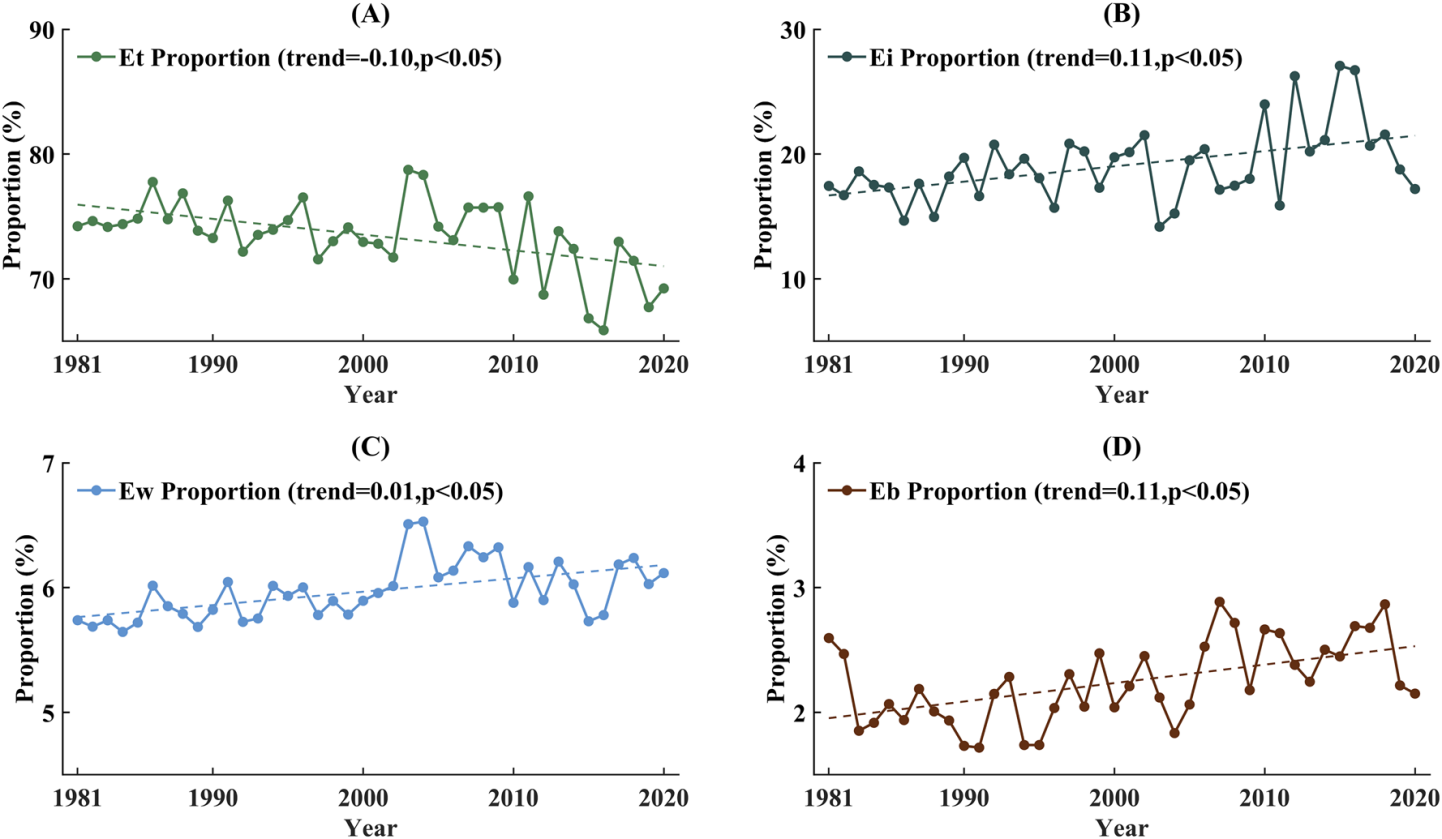
Proportion changes for components of ET_a_ between 1981 and 2020, (**A**) Et, (**B**) Ei, (**C**) Ew, (**D**) Eb.

Regarding the proportional changes in components, Fig. 8 demonstrates that the interannual variations in Ei, Ew, and Eb proportions are consistent with ET_a_. The trends of the Ei, Ew, and Eb ratios generally aligned with those of ET_a_. However, increases in Et and its proportion exhibited opposite trends, particularly after 2000. During the same period, the proportion of Et experienced a more pronounced decrease, whereas that of Ei showed a significant upward trend. The increase in Ei partially offsets the declining trend of Et; thus, ET_a_ continued to exhibit an increasing trend. Therefore, it can be assumed that the growth of ET_a_ in the Southeast River basin over the past four decades has mainly been attributed to vegetation, which needs to be considered when exploring the factors influencing ET_a_.

### Influencing factors in ET_a_ change

In general, ET_a_ changes in humid regions are mainly limited by energy conditions^10^, such as temperature or wind speed. The correlation coefficient between precipitation and ET_a_ was calculated to be only 0.1 (p = 0.27), which is a small correlation, so it was not analysed as an influencing factor^46^.

The results of the correlation analysis (Table 2) indicated that temperature, relative humidity, and vegetation were significant factors affecting ET_a_ variation in the Southeast River basin. This was supported by the strong correlations between ET_a_ and T (r = 0.76, *p* < 0.01), relative humidity (Rhu) (r = − 0.72, *p* < 0.01), and NDVI (r = 0.78, *p* < 0.01).

**Table 2:** Pearson’s correlation coefficient shows ET_a_ and the influencing factors in the Southeast River basin.

Factors	T	Win	Rhu	NDVI
1981–2020	0.76***	0.13	− 0.72***	0.78***

***Indicates significance at the 99% confidence level.

**Figure 9:**
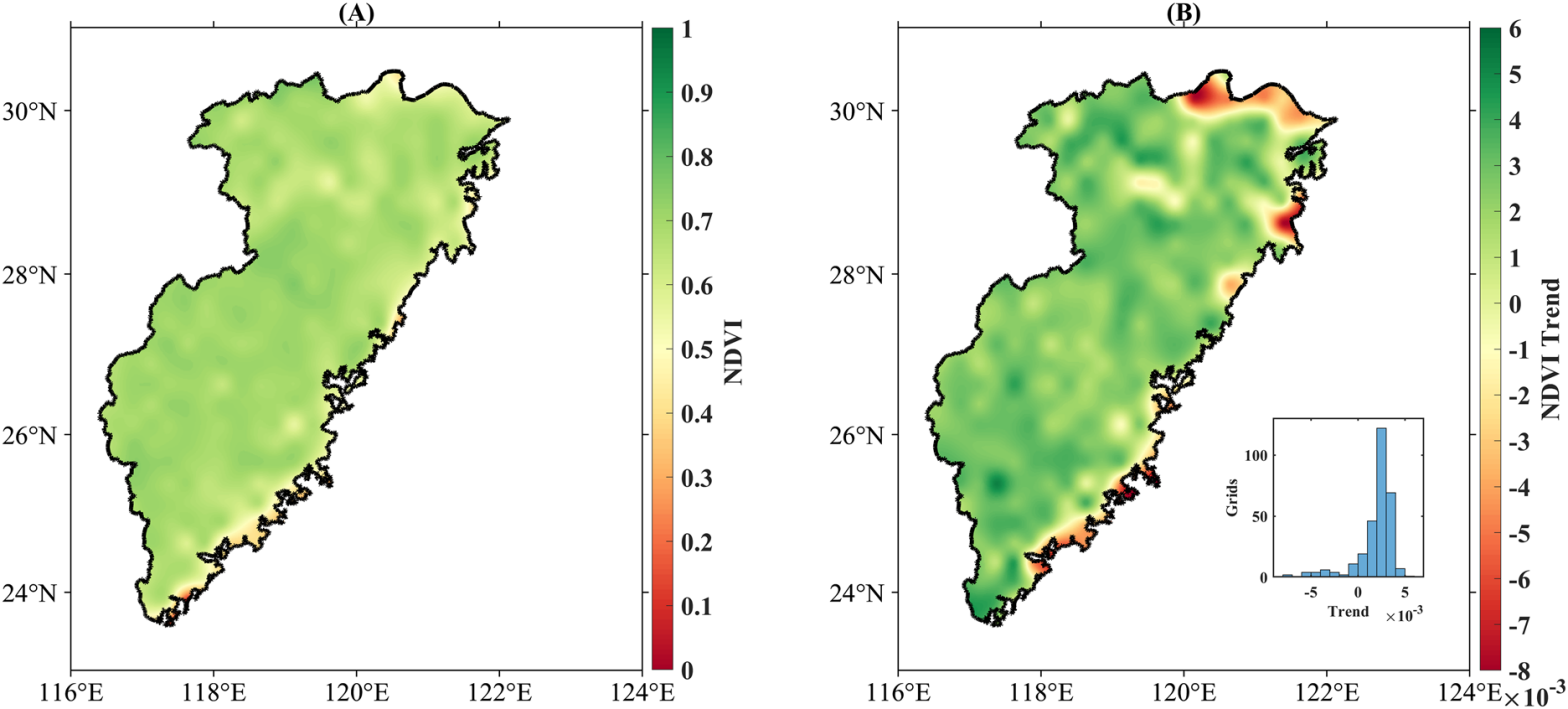
A spatial pattern of averaged NDVI index (**A**) and trend (**B**) over the Southeast River basin from 1981–2020. (We used MATLAB2021a to generate the figure, URL: https://ww2.mathworks.cn/products/matlab.html).

The contribution of influencing factors to changes in ET_a_ was calculated using a multiple regression model. The R^2^ of the regression equation was 0.89, indicating that the multiple regression model simulation was effective. A significant increase in T, Win, and NDVI occurred from 2001–2020 compared to 1981–2000, while Rhu experienced a notable decrease. This shift resulted in a marked increase in ET_a_ from 2001–2020. Table 3 indicates that T is the primary contributor to ET_a_, accounting for 47.93% of the total change, followed by Rhu at 40.45% and NDVI at 10.66%. These three factors have accounted for a total contribution of 99.11% to ET_a_ in the Southeast River basin since 2000. Although the contribution of NDVI was only 10.66%, its spatial changes (Fig. 9) exhibited a pattern similar to that of ET_a_ (Fig. 5).

**Table 3:** Contribution of each influencing factor to ET_a_ increase (%).

Factors	ET_a_	T	Win	Rhu	NDVI
T1: 1981–2000	0.25	0.33	0.41	0.81	0.34
T2: 2001–2020	0.79	0.69	0.49	0.37	0.62
Amount of Change	0.54	0.36	0.08	− 0.44	0.28
Regression coefficients		0.63	0.06	− 0.44	0.18
Actual contribution rate /%		47.93	0.96	40.45	10.66

### Relationship between changes in ETa and seasonal drought

**Figure 10:**
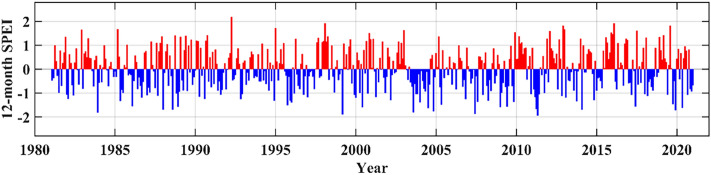
Monthly 12-month SPEI from 1981 to 2020.

The 12-month SPEI showed an increasing trend in the frequency of mid-droughts (SPEI < − 1.0) in the study area during the period 1981–2020 (Fig. 10). In 1981–2000, 30 months with SPEI < − 1.0 were recorded; in 2001–2020, 36 months with SPEI < − 1.0 were recorded. At the same time, SPEI showed a decreasing trend in most regions, indicating a drying trend, and only localised regions in the northeastern part showed an increasing trend in SPEI (Fig. S2). This suggests that spatial variation in SPEI has a good negative correlation with the variation in ET (Fig. 5).

**Figure 11:**
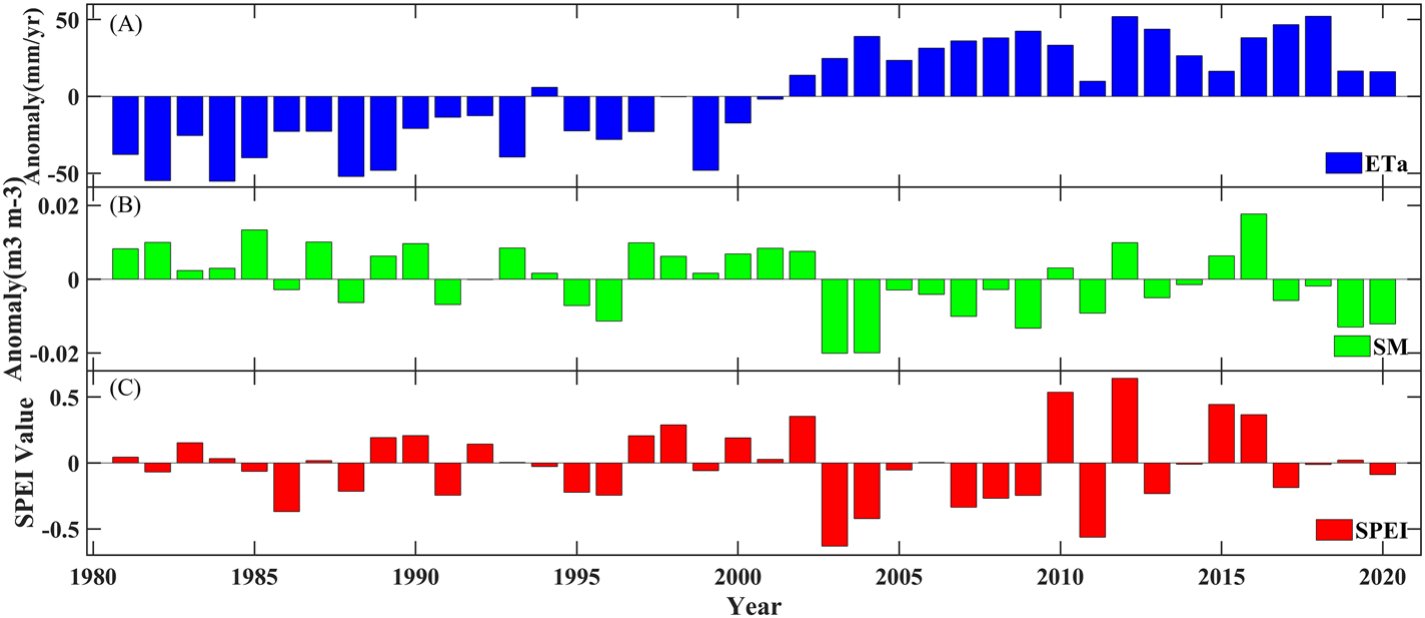
Changes in ET_a_ (**A**), SM (**B**), and SPEI (**C**) in the Southeast River basin from 1981 to 2020. The base period of ET_a_ and SM is 1981–2020.

ET_a_ plays a triggering role during drought events. The results depicted in Fig. 11 also demonstrate good correspondence between the variation in ET_a_ and alterations in soil moisture (SM) and SPEI. From 1981–2000, negative ET_a_ anomalies corresponded to positive soil moisture anomalies, whereas the SPEI changes remained relatively small, indicating a low incidence of drought. However, from 2001 to 2020, positive ET_a_ anomalies were accompanied by negative soil moisture anomalies, resulting in an escalating frequency of drought events. Therefore, ET_a_ changes are closely related to drought in the Southeast River basin.

Here, we selected two of the most severe drought years from 1981 to 2020 for a brief analysis (Figs. S3,S4). In spring 2003, there was a shift from a negative to a positive; however, at this time, there was more soil moisture and the drought was less severe. In 2011, ET_a_ did not change much but the spring drought was the most severe of the year. Compared to 2003, 2011 was less dry throughout the year but there was significant drought in the spring. This suggests that although there is a tendency for wetting in the Southeast River basins, droughts are more likely to be concentrated in one period and therefore may lead to more severe water shortages.

**Figure 12:**
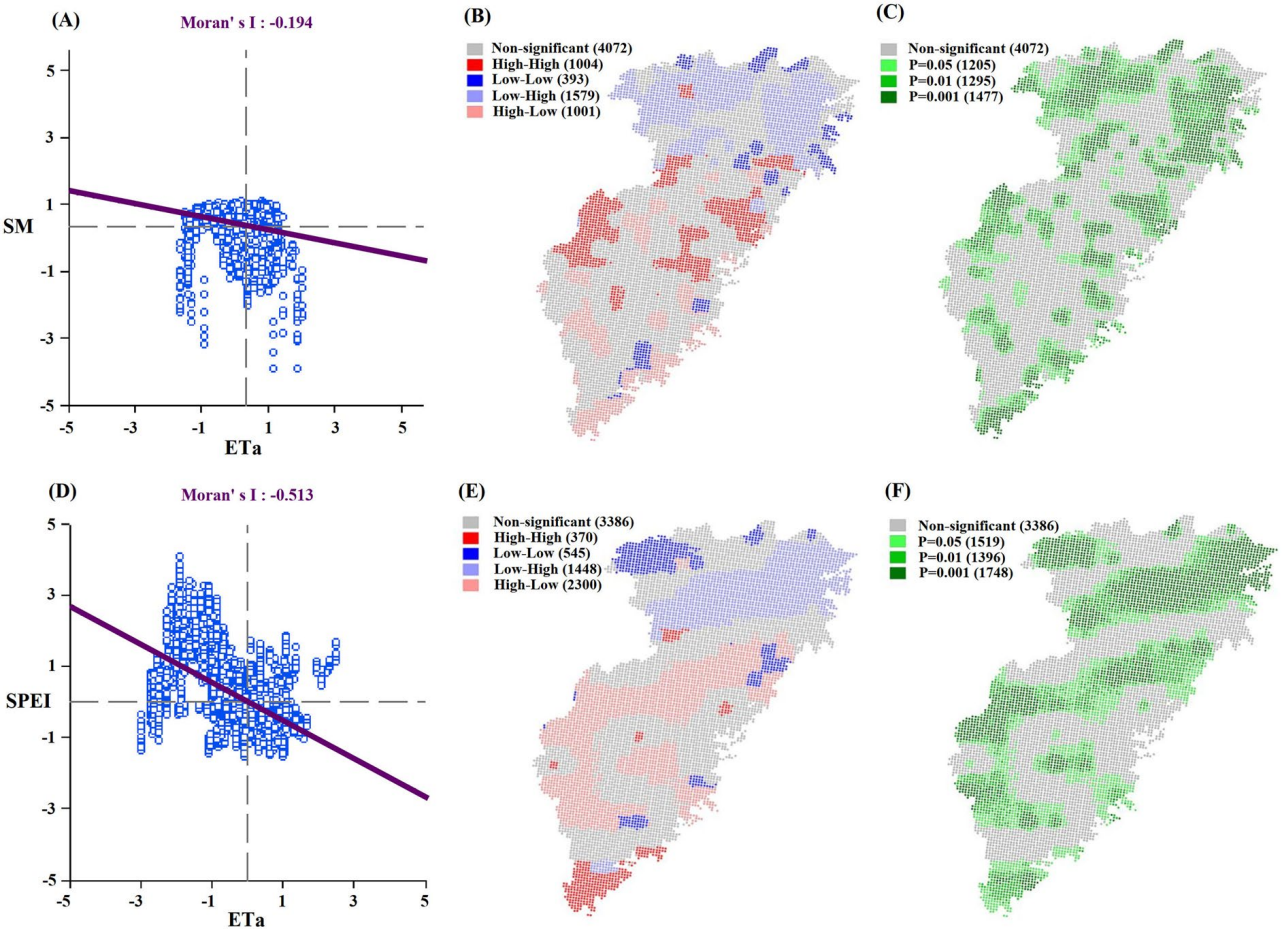
Spatial correlation analyses between ET_a_ and SM/SPEI in the Southeast River basin based on Moran’s index. Moran’s scatterplot (**A**), spatial clustering (**B**), and significance distribution (**C**) of ET_a_ and SM. Moran’s scatterplot (**D**), spatial clustering (**E**), and significance distribution (**F**) of ET_a_ and SPEI. (We used GeoDa (1.20.0.36) to generate the figure, http://geodacenter.github.io/download.html).

The spatial correlations between ET_a_, SM, and SPEI were analysed and the results are shown in Fig. 12. From 1981 to 2020, the Moran’s Index of ET_a_ and SM was − 0.194, showing a negative correlation (Fig. 12A). Regarding the cluster distribution (Figs. 12B,C), significant negative correlations occurred in the north (low–high correlation) and south (high–low correlation). Moran’s Index of ET_a_ and SPEI was − 0.513, indicating a highly significant negative spatial correlation (Fig. 12D). The cluster distribution (Fig. 12E,F) indicated that high-low and low–high markers occurred in most areas (the low–high correlation occurred in the northern part, and the high-low correlation was in the central and southern regions), proving that ET_a_ and SPEI are predominantly negatively correlated, suggesting that increased ET_a_ will lead to drought occurrence to a certain extent. Therefore, an increase in ET_a_ is an essential driving factor for drought in this region.

## Discussion

The drivers of ET_a_ variability are multifaceted and are influenced by various climatic factors. The ET_a_ changes in humid zones are primarily influenced by energy limitation^47,48^. For instance, among the 110 watersheds in southern China, temperature increase was identified as the dominant climatic factor contributing to the rise in ET_a_ in 104 watersheds^49^. As a typical humid subtropical region, the temperature variation in the Southeast River basin was closely correlated with changes in ET_a_. Over the last half-century, the temperature in the Southeast River basin has increased significantly, contributing nearly half of ET_a_ changes in the basin^50^. Rhu is a crucial factor affecting the variation of ET_a_. The findings in Table 2 demonstrate a significant negative correlation between relative humidity and ET_a_, as higher relative humidity levels led to lower saturated water vapour pressure differences, suppressing variations in ET_a_^51^. Rhu in the Southeast River basin showed a general decreasing trend during 1981–2020, which is consistent with the conclusion found by Lu that the relative humidity in eastern China (east of 100°E) generally decreased from 1987 to 2007^52^. Thus, the elevated growth in ET_a_ over the 2001–2020 period may have been partly a result of the apparent reduction in Rhu. Additionally, winning might be one of the factors that influence changes in ET_a_. Wind primarily affects ET_a_ by affecting the moisture conditions of the surface and higher wind speeds result in increased ET_a_^53^. Over the past 40 years, wind speed has increased slightly within the Southeast River basins, while ET_a_ has increased significantly in this region. Furthermore, the correlation coefficient between winds and ET_a_ was insignificant (Table 1), suggesting that changes in winds may have less influence on ET_a_ than other climatic factors.

In addition to climatic factors, vegetation had a significant effect on ET_a_. Vegetation change significantly influences changes in ET_a_ and variations in the proportion of its components^54,55^. The marked increase in urban land use within the Southeast River basin's northern and southeastern coastal regions between 1990 and 2015 is more closely aligned with the distribution of negative NDVI growth (Fig. 9). Being both the primary land-use type and exhibiting a significant growth trend (937 km^2^ increase)^16^, forested areas within the basin may account for the growth of the overall NDVI. An increase in vegetation will significantly increase vegetation transpiration and forest canopy interception, while concurrently leading to a decline in Eb. This decrease occurred because Eb accounted for a smaller proportion of the total ET_a_^56^. Therefore, future studies on ET_a_ influencing factors should focus on a quantitative analysis of the degree of contribution of climate and vegetation factors to ET_a_ variation, the contribution of multiple factors in different seasons of the year, and further investigate the influence of ET_a_ variation on the regional hydrological cycle and water storage.

By analysing 40 years of ET_a_ and SPEI, it was found that the annual scale of the Southeast River basin exhibited a gradual wetting trend, with a slow increase in both ET_a_ and SPEI. This may be attributed to other factors, such as the significant increase in vegetation cover within the basin, leading to increased Et and Ei^49^. These findings are consistent with those reported by Yang et al.^9^. From 1981 to 2020, the largest increase in ET_a_ within the Southeast River basin occurred in spring, followed by winter, summer, and autumn. Meanwhile, the SPEI displayed significant decreases during spring, smaller decreases during autumn, and an increasing trend throughout summer and winter. This suggests that drought conditions intensify in spring in the Southeast River basin. The observed trend, particularly the alteration in SPEI during spring, is consistent with the relationship with the trend in ETa during spring, as ascertained in the present study. In turn, this study also found a significant positive correlation between ET_a_ and SPEI through Moran's index; thus, a decline in SM and intensification of spring drought may occur within the Southeast River basin due to the persistent escalation of ET_a_ during springtime over recent years and amplified agricultural irrigation activities^57^. The Southeast River basin is economically developed and populated and the occurrence of droughts can affect the region's economic development and the ecological environment. Thus, the basin needs to strengthen its preparedness for seasonal droughts, such as spring droughts, to minimize the regional social and economic impacts of droughts when they occur. However, there are some limitations in this study, such as the resolution of the GLEAM product being 0.25°, which may be rough at the basin scale. In the future, other high-precision ET products based on the fusion of meteorological station data and multi-source remote sensing data should be considered to improve the accuracy of the analysis of ET elements and provide better guidance and support for water resource management and planning in the Southeast River basin.

## Conclusion

There was a significant upward trend in ET_a_ within the Southeast River basin from 1981–2020, with an increase rate of 2.51 mm/year (*p* < 0.05). Meanwhile, an abrupt change in ET_a_ occurred in 2000 as the average ETa showed an increase from 868.29 mm in 1981–2000 to 927.16 mm in 2001–2020. Seasonal differences in ET_a_ were substantial, with the highest total amount observed during summer and the most remarkable growth rate observed during spring. This increase was primarily due to contributions from spring and summer. The spatial distributions of seasonal and annual ET_a_ changes increased in most regions. The increase in ETa in the Southeast River basin was due to Et, Ei, Ew, and Eb, with Et (46.28%) and Ei (31.41%) being the dominant contributors.

Strong correlations were observed between ETa and T (*r* = 0.76, *p* < 0.01), Rhu (*r* = − 0.72, *p* < 0.01), and NDVI (*r* = 0.78, *p* < 0.01) in the Southeast River basin. Of these factors, temperature was identified as the dominant contributor (47.93%) to increased ET_a_ since 2000, followed by relative humidity (40.45%) and vegetation (10.66%).

There was good correspondence between the variation in ET_a_ and alterations in SM and SPEI. The Moran’s Index of − 0.513 and LISA showing high-low and low–high markers prove that ET_a_ and SPEI had a highly significant negative spatial correlation. Therefore, an increase in ET_a_ is an essential trigger of drought in this region.

### Supplementary Information

Supplementary Information.

## Data Availability

The raw data supporting the conclusions of this article will be made available by the authors, without undue reservation.
