# Supplementary material for: The influence of variations in actual evapotranspiration on drought in China's Southeast River basin
Source: Sci Rep. 2023 Dec 4;13:21336. doi: 10.1038/s41598-023-48663-8 (PMC10696048; doi:10.1038/s41598-023-48663-8)

**Supplementary Material**

**The influence of variations in actual evapotranspiration on the drought in China's Southeast River basin**

Sheng Hong^1,2,3,4^, Haijun Deng^1,2,3,4^*, Zhouyao Zheng^1,2,3,4^, Yu Deng^1,2,3,4^, Xingwei Chen^1,2,3,4^, Lu Gao^1,2,3,4^, Ying Chen^1,2,3,4^ and Meibing Liu^1,2,3,4^

^1^ Institute of Geography, Fujian Normal University, Fuzhou 350117, China.

^2^ Fujian Provincial Engineering Research Centre for Monitoring and Assessing Terrestrial Disasters, Fujian Normal University, Fuzhou 350117, China.

^3^ Key Laboratory of Humid Subtropical Eco-geographical Processes of Ministry of Education, School of Geographical Sciences, Fujian Normal University, Fuzhou 350117, China.

^4^ Fujian Provincial Key Laboratory for Plant Eco-Physiology, Fujian Normal University, Fuzhou 350117, China.

* Correspondence: Haijun Deng; e-mail: denghj@fjnu.edu.cn

**Supplementary Figure S1.** The M-K mutation test in the Southeast River basin from 1981–2020.
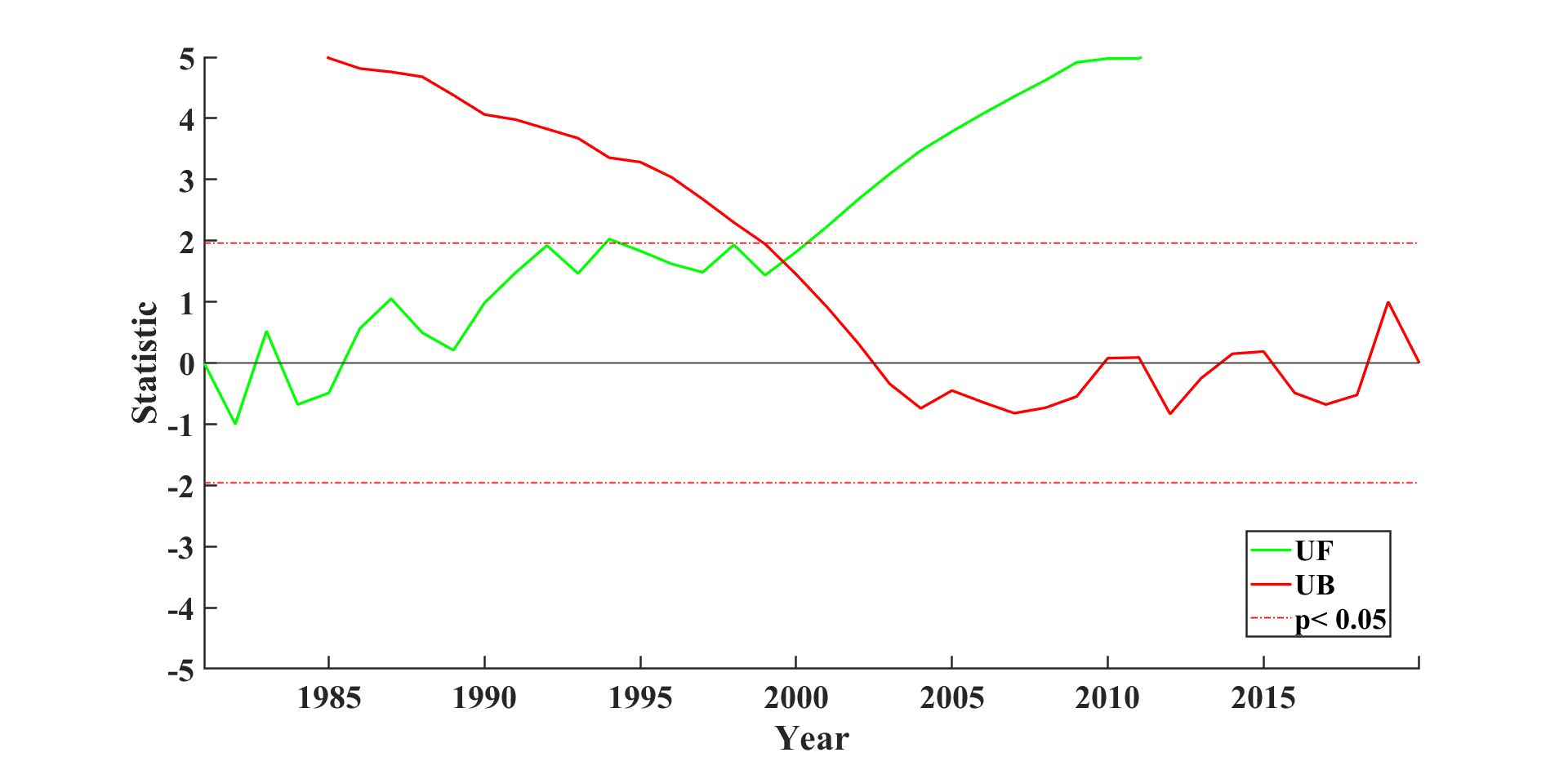


**Supplementary Figure S2.** Spatial distribution of annual changes in 12-month SPEI index from 1981–2020.


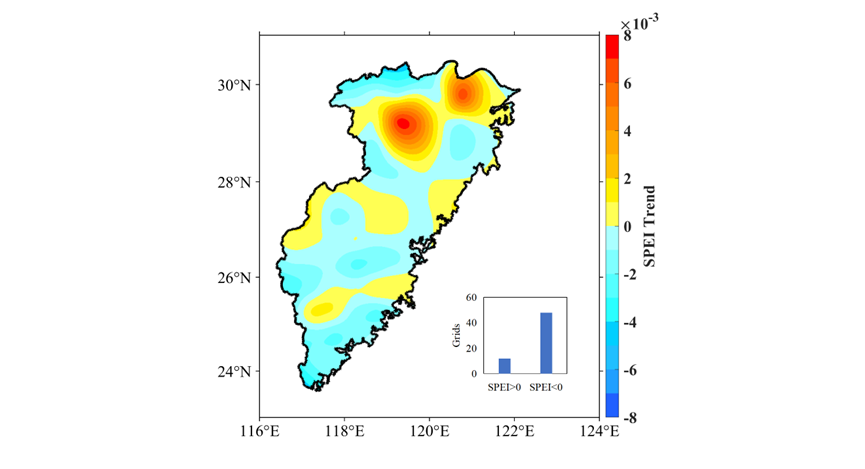


**Supplementary Figure S3.** Bar plot of monthly ETa (A), SM (B), and SPEI (C) in 2003. The base period of ETa and SM is 1981–2020.


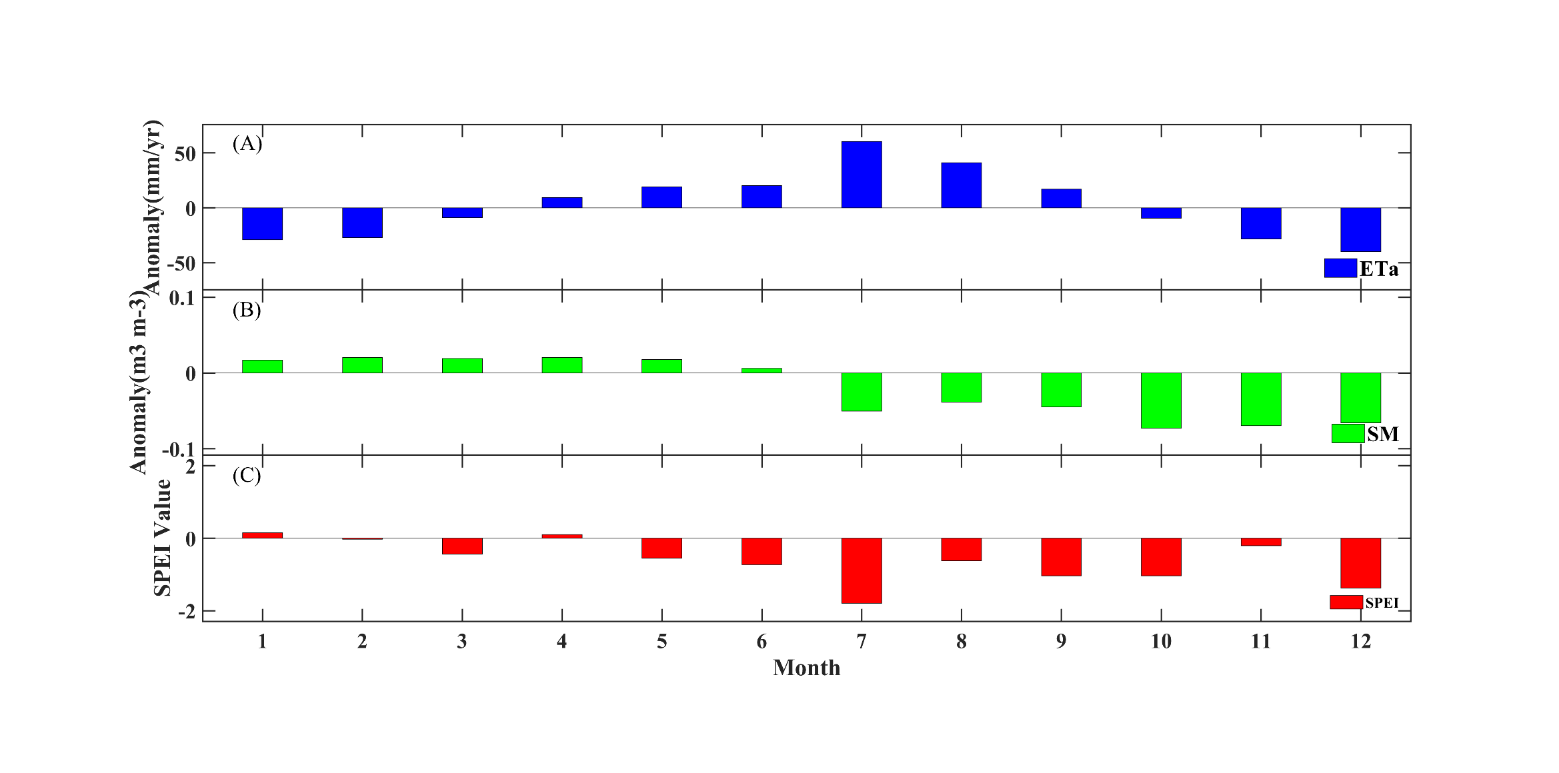


**Supplementary Figure S4.** Like Figure S3, this figure depicts the data for the year 2011.


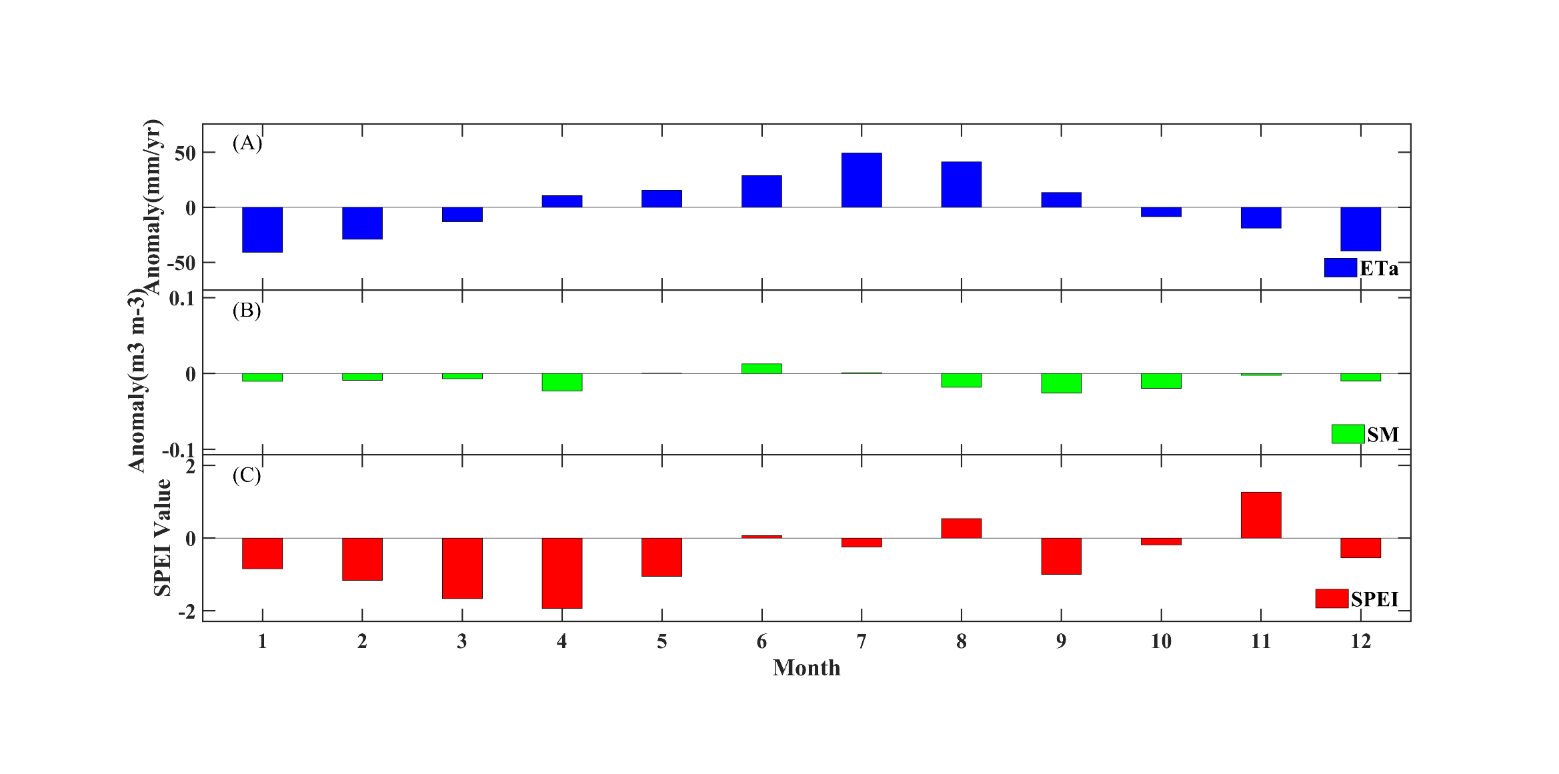

Supplement: Supplementary file 1 — Supplementary Information. [file 41598_2023_48663_MOESM1_ESM.docx]
